# Detect Cytochrome C Oxidase- and Glutathione-S-Transferase-Mediated Detoxification in a Permethrin-Resistant Population of *Lygus lineolaris*

**DOI:** 10.3390/toxics11040342

**Published:** 2023-04-04

**Authors:** Yu-Cheng Zhu, Yuzhe Du, Jianxiu Yao, Xiaofen F. Liu, Yanhua Wang

**Affiliations:** 1 United States Department of Agriculture, Agricultural Research Service, Jamie Whitten Delta States Research Center (USDA-ARS-JWDSRC), Stoneville, MS 38776, USA; 2 Department of Entomology, Kansas State University, Manhattan, KS 66506, USA; 3 Zhejiang Academy of Agricultural Sciences, Hangzhou 310004, China

**Keywords:** gene regulation, microarray, pathway, P450, esterase, metabolic, cross resistance

## Abstract

Frequent sprays on cotton prompted resistance development in the tarnished plant bug (TPB). Knowledge of global gene regulation is highly desirable to better understand resistance mechanisms and develop molecular tools for monitoring and managing resistance. Novel microarray expressions of 6688 genes showed 3080 significantly up- or down-regulated genes in permethrin-treated TPBs. Among the 1543 up-regulated genes, 255 code for 39 different enzymes, and 15 of these participate in important pathways and metabolic detoxification. Oxidase is the most abundant and over-expressed enzyme. Others included dehydrogenases, synthases, reductases, and transferases. Pathway analysis revealed several oxidative phosphorylations associated with 37 oxidases and 23 reductases. One glutathione-S-transferase (GST LL_2285) participated in three pathways, including drug and xenobiotics metabolisms and pesticide detoxification. Therefore, a novel resistance mechanism of over-expressions of oxidases, along with a GST gene, was revealed in permethrin-treated TPB. Reductases, dehydrogenases, and others may also indirectly contribute to permethrin detoxification, while two common detoxification enzymes, P450 and esterase, played less role in the degradation of permethrin since none was associated with the detoxification pathway. Another potential novel finding from this study and our previous studies confirmed multiple/cross resistances in the same TPB population with a particular set of genes for different insecticide classes.

## 1. Introduction

The tarnished plant bug (TPB), *Lygus lineolaris*, is a polyphagous insect and can easily be found year-round on a variety of crops in Mid-south US. Although transgenic Bt crops are effective against many insect pests, the TPB is still the most economically important pest in Mid-south cotton growing areas [1]. Damage caused by feeding TPBs leads to malformed cotton bolls with dark lesions, resulting in the shedding of squares, small bolls, stunted plants, aborted terminals, boll deformation, lint staining, and yield loss [2]. Currently, chemical control with synthetic insecticides is the primary control method for this pest. More than forty insecticides are currently recommended by extension specialists in the United States for the chemical control of row crop insects [3,4,5]. Many insecticides have been used on cotton for controlling TPB, bollworm, and a range of other pests, including pyrethroids, organophosphates, carbamates, neonicotinoids, and novel insect growth regulators [3].

Permethrin is a neurotoxic synthetic pyrethroid insecticide. It acts on the nervous system of insects, interfering with sodium channels to disrupt neuron function, causing muscles to spasm, culminating in paralysis and death [6,7]. Long-term use of chemical insecticides for TPB control gradually decreases the efficacy of insecticides, and resistance to pyrethroids has been found in many TPB populations in the mid-south [8,9,10]. Since the first report of pyrethroid resistance in a tarnished plant bug field population high enough to cause a control failure in cotton [11], very little research has been conducted to determine biochemical and molecular mechanisms for resistance development in the natural ecosystem before the 2000s. Research has concentrated on knockdown resistance and has provided evidence that mutations in sodium channels are the primary resistance mechanism [12]. However, many researchers have linked mechanisms of pyrethroid resistance with metabolic detoxification enzymes, such as P450 monooxygenases, esterases, and glutathione S-transferases [13,14,15]. Zhu et al. [16] and Zhu and Luttrell [17] found that TPB populations were able to develop multiple and cross resistance to different insecticides (classes). If resistance is left unchecked, the cost due to yield loss, control cost increase, and environmental contamination could be enormous.

Quantitative real-time PCR (qrt-PCR) is a common method for studying gene regulation and insecticide resistance mechanisms by measuring whether relevant gene expressions are altered [18]. However, the method is inefficient, and no more than 96 genes can be examined at a time. As an alternative, microarray technology is a powerful tool for examining the expression of thousands or even millions of genes at once [19]. To understand how gene regulation and biochemical processes influence insecticide susceptibility, we took advantage of robust microarray technology in this study to analyze 6688 gene expressions simultaneously in a TPB population collected from a cotton field and subsequently selected with permethrin. A laboratory colony unexposed to any pesticide was used as a side-by-side control. To identify genes participating in detoxification pathways, Blast2go annotation, and enzyme KEGG pathway analyses were also applied to reveal the functions of major significantly up- and down-regulated genes. By using these novel microarrays and powerful analytical tools, we were able to identify a set of metabolic genes relevant to the detoxification of insecticides, thus expanding our ability to effectively monitor and manage permethrin resistance in TPB, a great step forward in our understanding of resistance in pest insect populations.

## 2. Materials and Methods

### 2.1. Insect Laboratory Colony and Field Population

A laboratory colony (LLMCK) was originally provided by Kathy Knighten and Fred Musser at Mississippi State University. The colony, established in 2005 from a collection of tarnished plant bug from the Mississippi Delta area, has been maintained on an artificial diet [20] without exposure to insecticide. Wild adults (collected from fleabane [*Erigeron* spp.] and ryegrass [*Lolium* spp.]) are introduced into this colony in the spring of each year to enhance genetic diversity and colony vigor. This colony was used as a standard susceptible strain.

During 2011, many field populations were collected and subjected to discriminating dose assays using permethrin (Arctic 3.2 EC, 36.8% a.i., Winfield Solution, LLC, St. Paul, MN, USA) at 145 mg/L Arctic formulation. Bugs were treated with a modified spray tower, and mortality was recorded after 48 h. To prepare samples for microarray analysis, a field population was collected in July 2011 from a cotton field north of Stoneville, Mississippi. Permethrin-selected bugs (Arct2175FF) were from a permethrin treatment of the field population. Approximately 300 bugs were collected and selected with Arctic 3.2 EC at 2175 mg/L (or 800.44 mg a.i./L permethrin), which was 15-fold (resistance ratio = 15-fold) higher than the LC_50_ of LLMCK. Adults were held in a 2.5-ga bucket covered with fine (10 grids/cm) net cloth at the top and bottom. Two mL of permethrin solution (4× higher volume than that used for the LC_50_ test) was used to treat the cage thoroughly from the top by using a modified Potter Spray Tower. The sprayer was set at 7.5 psi with a spray distance of 30.5 cm to ensure uniform deposition of insecticide mist on the inner surface of the container, green beans (supplied as food for TPBs), and bugs. Treated bugs stayed on permethrin-treated green beans for 48 h, and survivors (showing normal viability) were collected and stored at −80 °C for microarray analysis.

### 2.2. Preparation of cDNA Sequences for Microarray Expression Chips

Total RNA was prepared using TriZol reagent (Invitrogen, Carlsbad, CA, USA). mRNA was purified from total RNA using the NucleoTrap mRNA purification kit (BD Bioscience Clontech, Palo Alto, CA, USA). The Creator Smart cDNA Library Construction Kit (BD Bioscience Clontech) was used for cDNA library construction by following the manufacturer’s instructions and modified protocols described by Zhu et al. [21]. Approximately 30,000 clones were obtained and sequenced with an M13 forward primer on an ABI 3730XL sequencer. cDNA sequences were assembled using Lasergene software (DNAStar, Madison, WI, USA) and subjected to a similarity search for putative identity against GenBank protein and nucleotide databases (http://blast.ncbi.nlm.nih.gov/Blast.cgi (accessed on 1 April 2023)). cDNA sequences were submitted to Roche NimbleGen (Roche NimbleGen, Inc., Madison, WI, USA) for the production of 72K gene expression chips in 4-plex format. A 60-bp specific oligonucleotide was designed and synthesized as a probe. Approximately 35,000 probes (an average of 5 probes per cDNA) were synthesized and printed on each gene expression chip.

### 2.3. Acquiring Microarray Data

Roche NimbleGen gene expression chips were used to compare global gene expression between permethrin-selected (Arct2175FF) and susceptible (LLMCK) strains of TPB. Microarray analysis was processed using standard NimbleGen array protocols. Total RNA was extracted from adults using TriZol reagent (Invitrogen). Double-strand cDNAs were synthesized by using the SuperScript Double-Stranded cDNA Synthesis Kit (Invitrogen) according to the manufacturer’s protocols. Double strand cDNA samples were labeled with the One-color DNA Labeling Kit and hybridized to the microarray chips. Microarray data were acquired according to NimbleScan v.25 User’s Guide through Florida State University’s Microarray processing facility. Three arrays (3 replicates) of 72K NimbleGen expression chips were processed for each sample.

### 2.4. Analysis of Microarray Data

After gene expression data were obtained from 4 × 72 K array processing, ArrayStar^®^ software (DNAStar, Inc., Madison, WI, USA) was used to analyze and compare microarray data between LLMCK and Arct2175FF. Expression data were log2-transformed and normalized through quantile normalization [22]. The data were analyzed using classical parametric statistics. *p*-values were calculated using a Modified *t*-test. A fold-change cutoff of 2 and *p*-value threshold of 0.05 were used to determine significantly differential gene expression.

After microarray data were processed, up-regulated and down-regulated genes (cDNAs) were separately subjected to sequence annotation and pathway analysis using step-by-step processing of Blastx, mapping, annotation, enzyme code, and KEGG analysis protocols provided by Blast2go (https://www.blast2go.com (accessed on 1 April 2023)).

## 3. Results

### 3.1. Scatter-Plot Comparison of Gene Expression Levels between LLMCK and Arct2175FF

**Figure 1:**
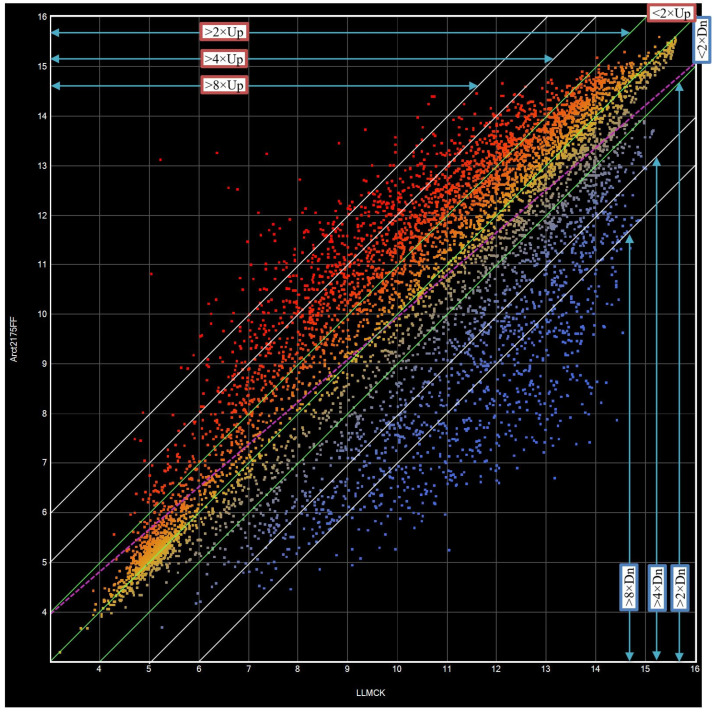
Analysis of microarray data and comparison of 6688 gene expression levels between susceptible (LLMCK) and permethrin-selected (Arct2175FF) tarnished plant bugs using ArrayStar software. Scatter-plot comparison of 6688 gene expression levels between LLMCK and Arct2175FF. The mini squares of the scatter plot in the upper left corner represented up-regulated genes, and the squares in low right corner represented down-regulated genes. Squares above line 2 × Up and below line 2 × Dn represent up- and down-regulated genes by 2-fold; Squares above line 4 × Up and below line 4 × Dn represent up- and down-regulated genes by 4-fold; Squares above line 8 × Up and below line 8 × Dn represent up- and down-regulated genes by 8-fold.

A total of 7446 unique contigs and singletons were obtained from cDNA library sequencing, with 6688 genes showing valid expression values from hybridization to labeled probes (Double-Stranded cDNAs of permethrin-resistant Arct2175FF and susceptible LLMCK bug samples). Figure 1 was from scanned signals of hybridized gene chips of Arct2175FF that were log2 converted, normalized, and plotted against the corresponding signals of the susceptible (LLMCK) strain. The plot (Figure 1) shows distributions of 6688 gene expression levels. Each tiny square represents a unique gene expression ratio between Arct2175FF and LLMCK strains. Gene expression levels of the two strains showed a linear correlation (R^2^ = 0.721). Statistics show the expression levels of 3080 genes in permethrin-selected (Arct2175FF) bugs were up- or down-regulated by >2-fold. Among those 3080 genes, 1364 were up/down-regulated by >4-fold, and 539 were up/down-regulated by >8-fold. Another 2622 genes were up/down-regulated by less than 2-fold (1205 up and 1417 down) (Figure 1). The expression levels of the remaining 986 genes studied were not significantly different between Arct2175 and LLMCK.

### 3.2. Identity of Up- and Down-Regulated Genes

Among 1543 up-regulated (>2 fold) genes, 255 code for 39 enzymes. Based on function and participation in important pathways involving metabolic detoxification and resistance development, Table 1 lists contig names, sequence length, up-regulation levels (fold), coded enzymes, and other parameters of 187 enzyme-coding cDNAs. Fifteen different metabolic enzymes are encoded by these 187 cDNAs; several of these enzymes are encoded by multiple cDNA sequences, including 64 cDNAs for oxidases, 49 cDNAs for dehydrogenases, 28 cDNAs for synthases, 11 cDNAs for reductases, 11 cDNAs for transferases, 7 cDNAs for esterases, 5 cDNAs for glutathione S-transferases, and four or fewer cDNAs for ATPase, cytochrome P450 monooxygenases, phosphatases, phosphodiesterase, thioesterases, ATP sythetases, and transcriptases. Some synthases may include ATP synthases, ATPases, and synthetases (Table 1). Another 24 enzymes coded by 68 cDNAs were not included in Table 1; they are amidase, amylase, carboxypeptidase, cathepsin, cysteine protease, dioxygenase, dismutase, exonuclease, glucosidase, helicase, hydrolase, integrase, isomerase, kinase, ligase, lipase, lyase, myrosinase, nuclease, peptidase, peptidase, polygalacturonase, polymerase, and protease.

There were 386 enzyme-coding genes showing significantly reduced (>2-fold) expression levels, coding for 53 enzymes. By considering the importance of their functions and involvements in metabolic detoxification pathways, contig names, sequence length, down-regulation levels (fold), coded enzymes, and other parameters of 114 enzyme-coding cDNAs are listed in Table 2. These genes encode 13 important metabolic enzymes. Several of the enzymes are encoded by multiple cDNA sequences, including 26 cDNAs for dehydrogenases, 19 cDNAs for ATP synthases or ATPases, 18 for transferases, 11 for P450 monooxygenases, 9 for reductases, 8 for hydrolases, 7 for oxidases, 6 for esterases, 5 for GSTs, 3 for reductases/peptidases, one for peroxidase, and one for phosphate synthase (Table 2). In addition, another 40 encoded enzymes may be indirectly involved in some metabolic processes (not included in Table 2). They are aldolase, amylase, anhydrase, carboxypeptidase, cathepsin, chitinase, cysteine peptidase, cysteine protease, decarboxylase, deoxyribonuclease, desaturase, dioxygenase, dismutase, enolase, epimerase, fucosidase, glucosidase, glyoxalase, helicase, hexosaminidase, hydroxylase, isomerase, kinase, ligase, lipase, lyase, nucleotidase, ovochymase, peptidase, phosphatase, phosphodiesterase, polygalacturonase, polymerase, protease, RNase H, synthetase, thioesterase, transaminase, translocase, and trypsin. Several digestive-related enzymes are encoded by a large number of cDNA sequences, and their gene expression levels were significantly down-regulated in permethrin-treated bugs. These enzymes included 41 cDNAs for cathepsins, 41 cDNAs for proteases, 14 cDNAs for polygalacturonases, and 6 cDNAs for trypsins.

### 3.3. Annotation and Functional Analysis of Up-Regulated Genes in Arct2175FF

**Figure 2:**
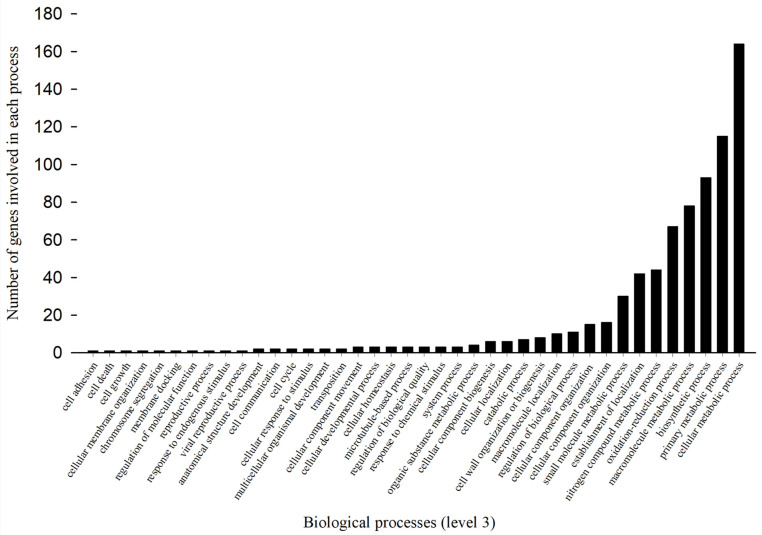
Variable number of up-regulated genes associated with each biological process in permethrin-selected tarnished plant bug.

**Figure 3:**
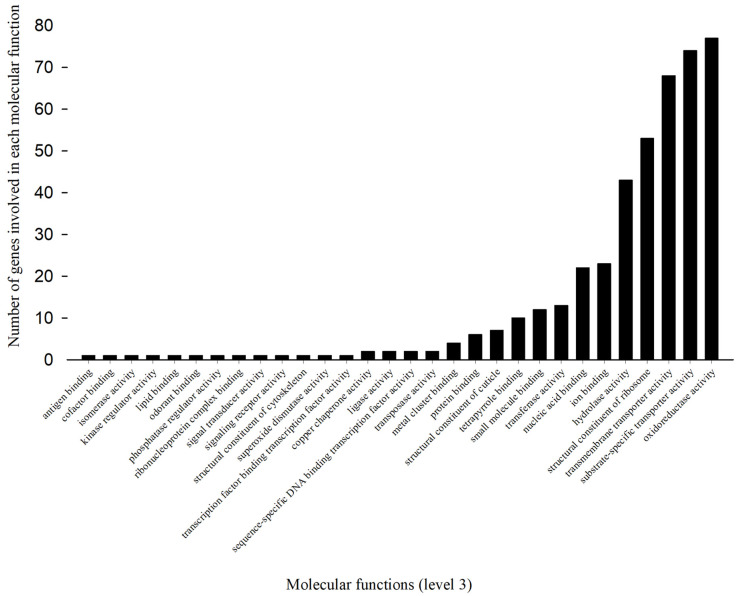
Variable number of up-regulated genes associated with each molecular function in permethrin-selected tarnished plant bug.

Reruns of Blast2go mapping, annotation, and KEGG analyses of 1543 up-regulated (≥2-fold) cDNAs revealed a large number of genes involved in biological processes (Figure 2) and molecular functions (Figure 3).

#### 3.3.1. Gene Regulation in Biological Processes

Annotation with Blast2go showed that 759 up-regulated genes were involved in 40 biological processes in Arct2175FF at GO level 3 (Figure 2). These are cumulative numbers of the genes for each biological process and are higher than the actual number of genes, indicating some genes had multiple functions. Among the 40 biological processes at GO level 3, cellular metabolic, primary metabolic, biosynthetic, macromolecule metabolic, and oxidation-reduction processes seemed to be particularly important, as indicated by the participation of higher numbers (164, 115, 93, 78, and 67, respectively) of genes in these processes than in other biological processes and the fact that metabolic detoxification is a major mechanism in insecticide resistance development. Other affected metabolic processes are nitrogen compound metabolism (44 genes) and small-molecule metabolism (30 genes); 24 of these 40 biological processes involved less than 4 up-regulated genes (Figure 2).

#### 3.3.2. Gene Regulation in Molecular Function

Annotation with Blast2go showed that at level 3, 433 up-regulated genes were influenced by permethrin treatment. These up-regulated genes were involved in 30 molecular functions in Arct2175FF (Figure 3). The most enhanced five molecular functions, oxidoreductase activity, substrate-specific transporter activity, transmembrane transporter activity, structural constituent of ribosome, and hydrolase activity, were associated with 77, 74, 68, 53, and 43 up-regulated genes, respectively.

### 3.4. Gene Regulation Influencing Metabolic Pathways in Arct2175FF

KEGG analyses of 1543 up-regulated genes (>2-fold) identified 165 up-regulated specific genes and their involvements in 27 specific pathways. In Table 3, 71 contig names of the cDNAs are listed for 9 important pathways potentially associated with metabolic detoxification and resistance development to permethrin in TPB. Genes involved in another 18 pathways, such as nitrogen, starch, and sucrose metabolisms, etc., are not included in Table 3. Oxidase and reductase (H+-translocating) (ec:1.6.5.3) genes are the primary up-regulated genes for the oxidative phosphorylation pathway (37 and 23 genes, respectively). A few dehydrogenase, reductase (ec:1.10.2.2), ATPase, and diphosphatase genes were also found to be associated with this pathway. The gene LL_2258 (GST) participates in three pathways, i.e., glutathione metabolism, drug metabolism—cytochrome P450, and metabolism of xenobiotics by cytochrome P450 (Table 3).

Analyses of 1537 down-regulated genes (>2-fold) identified 256 specific genes involving 147 pathways. In Table 4, 32 contigs containing cDNAs are listed to represent 12 pathways potentially associated with metabolic detoxification and resistance to permethrin in TPB. Another 135 pathways, such as nitrogen metabolism (oxidases and reductases), pyruvate metabolism (dehydrogenases, reductases, kinases), the citrate cycle (dehydrogenase, hydratase, ligases), etc., were affected but were not included in Table 4. Down-regulation of 12 ATPase genes made the phosphorylation pathway the most heavily influenced pathway by permethrin. LL_6284, coding for a GST, is associated with three detoxification pathways: glutathione metabolism, drug metabolism, and metabolism of xenobiotics (Table 4); however, LL_6284 was significantly down-regulated in Arct2175FF. LL_2553, a down-regulated phosphoribosyltransferase gene, also participates in drug metabolism. Large numbers of cathepsin (41), lipase (9), polygalacturonase (14), protease (41), and trypsin (6) genes were among the 1537 significantly down-regulated genes. In contrast, 11 of the 12 P450 but only 7 of the 71 oxidase genes were significantly down-regulated in Arct2175FF. In addition, 24 vitellogenin and 9 RP45 eggshell genes were significantly down-regulated in Arct2175FF.

## 4. Discussion

In this study, microarray analysis was applied to quantify expressions of 6688 gene transcripts (simply called genes) simultaneously. Most of the 6688 cDNAs obtained using an ABI 3730XL sequencer were probably single reads (singletons). Five 60-bp oligonucleotides were designed as five probes (replicates) from different regions of each cDNA and printed on a gene chip. For microarray processing, we used three gene chips as three replications for each permethrin-treated sample and control sample. Therefore, each expression signal was averaged from 15 separate probe signals. Increased replicates and replications ensured data reliability which was higher than that of previously published data obtained from only one or two chips per sample. Microarrays were commonly used between 2002 and 2013 for the analysis of gene expression. Some hybridization data collected from nylon membranes dotted with less than 100 known cDNAs were also called microarray analysis. Next-generation sequencers facilitated the capacity of microarrays to analyze twenty thousand or more gene expressions at once [23,24,25]. Surprisingly, our microarray analysis detected greater numbers and diversity of detoxification genes than those microarrays using higher capacity chips (20,000 genes). Similarly, the popular and more recent (2015-present) RNA-Seq technology has been used to quantify pyrethroid resistance-related transcript expression against a larger gene pool [26,27,28]. However, RNA-Seq has not significantly increased knowledge of transcriptional expression patterns in either variety or number of pyrethroid resistance genes detected compared to microarrays. Furthermore, a novel mechanism of (large number) cytochrome c oxidase-mediated, along with a GST-mediated, detoxification as the major mechanism in permethrin-resistant TPB was detected in this study using microarray, which has not been detected using RNA-Seq or microarray even with higher capacity gene chips [26,27,28].

Functional analyses indicated that large numbers of up-regulated genes participated in 40 biological processes and 30 molecular functions. Of these, two biological processes, cellular metabolism and primary metabolism, are carried out by more than 100 up-regulated genes in permethrin-selected TPB. Molecular function analyses showed this trend in five molecular functions as well. Oxidoreductase activity, substrate-specific transporter activity, transmembrane transporter activity, structural constituent of ribosome, and hydrolase activity were the five most enhanced molecular functions in permethrin-treated TPBs. Oxidoreductase activity (pumps protons across the inner membrane of mitochondria or the plasma membrane [29]) is the most enhanced molecular function (Figure 3), which is catalysis of an oxidation-reduction reaction (oxidative phosphorylation [30] in Table 3) by oxidases, dehydrogenases, hydroperoxidases, and oxygenases. Therefore, both functional and pathway analyses (see below) indicated that oxidases reductases, dehydrogenases, ATPases, and diphosphatases were consistently associated with and facilitated pyrethroid detoxification [31] and resistance development in TPB.

By using blast2go and other molecular tools, we identified 256 significantly up-regulated genes that code for a variety of enzymes. In total, 187 genes code several functionally important enzymes that regulate 17 physiologically essential pathways. Nine of these pathways are associated with oxidative phosphorylation and metabolism of drugs and xenobiotics (i.e., detoxification), comprised of oxidases, reductases, dehydrogenases, ATPases, transferases, and cytochrome P450 monooxygenases. After frequent exposures to insecticides, some insect populations may evolve resistance to those insecticides via gene mutation [32,33] and [34]/or enhanced gene expressions [35].

By using microarray analysis, we found that cytochrome c oxidase genes are the most over-expressed enzyme genes in permethrin-treated TPB (Arct2175FF). Sixty-four oxidase genes were identified that code for at least 8 different oxidases from subunits 1 to 10 except subunits 4 and 5. Cytochrome c oxidase subunit 3 is the most abundant oxidase in permethrin-treated TPB, followed by subunits 7 and 2. Over-expression of oxidase subunits 3 and 1 was reportedly responsible for resistance development in many insects [36,37,38]. However, not all oxidase genes were up-regulated. Seven oxidase genes were found to be significantly down-regulated in permethrin-resistant TPB, including glutathione peroxidase-like, cytochrome c oxidase subunits 5a and 5b, cytochrome c oxidase assembly protein cox15, fad-linked sulfhydryl oxidase alr-like, and spermine oxidase. None of these down-regulated oxidase genes belong to the subunits (1–3 and 6–10) of up-regulated oxidase genes, indicating some oxidase genes are functionally diverged and are differentially regulated in permethrin-resistant TPB. In addition, oxidase subunit 4, coded by LL_1380, showed 1.195-fold down-regulation (<2-fold and therefore not significant). Molecular phylogeny analysis showed that TPB oxidase genes are relatively diverse: six subgroups have four or more members, and three have two members. Another 7–11 members’ positions have not been clearly resolved (phylogeny tree not included). Hence, this study profiles the gene expression of 11 subunits (1–10 and 15) of cytochrome c oxidase, significantly extending our knowledge of the involvement of these oxidase genes, particularly the overexpressed subunits 1–3 and 6–10 in permethrin-treated TPBs. These data will greatly facilitate the development of specific biomarkers and molecular control strategies for future studies.

Previously we used the same gene chips to analyze differences in gene expression between the susceptible colony LLMCK (also used in this study) and an imidacloprid-resistant population Im1500FF [17]. The Im1500FF TPBs were collected from the same location as Arct2175FF (Feather Farm) and treated with 1500 mg/L imidacloprid formulation (Advise 2FL). Only one oxidase (LL 547, subunit 1) gene was up-regulated in Im1500FF compared with 64 in Arct2175FF. Incidentally, LL 547, subunit 1 was significantly down-regulated in Arct2175FF. A total of 3 oxidase genes were significantly down-regulated in Im1500FF. Of these, LL_963 (subunit 7a), down-regulated in Im1500FF, was up-regulated in Arct2175FF. The other two, LL_791 (subunit 5a) and LL_5776 (spermine oxidase), were down-regulated in both Im1500FF and Arct2175FF. The data from comparison of the same population treated with different insecticides, imidacloprid (a neonicotinoid) for Im1500FF and permethrin (a pyrethroid) for Arct2175FF, indicated that cytochrome oxidase genes are predominantly associated with permethrin metabolic detoxification/resistance development, while P450 monooxygenase and esterases are the major genes for imidacloprid resistance. Therefore, it is clear that the same population had developed resistance to multiple insecticides and cross resistance to different insecticide classes (have different modes of action [17]) with different/unique sets of genes for different insecticides (classes) (see below).

Most resistance development is associated with over-expression of genes encoding metabolic detoxification enzymes, such as esterase, glutathione S-transferases (GST), and cytochrome P450 monooxygenase (P450) genes [39]. Cytochrome P450 monooxygenases (P450) catalyze the oxidation of organic substances to fulfill many important tasks, from the synthesis, degradation, and metabolic intermediation of lipids, ecdysteroids, and juvenile hormones to the metabolism of xenobiotics [40]. P450 genes play a central role in the adaptation to plant chemicals and the development of resistance to pesticides. It is well established that many cases of metabolic resistance to insecticides are the result of elevated levels of P450 [41]. Only one P450 gene (LL_3359) was significantly up-regulated, but it did not participate in any important pathway (Table 3); 11 P450 genes were significantly down-regulated, but again none of the 11 P450s was involved in an important pathway in Arct2175FF, with the exception of the P450 encoded by significantly down-regulated LL_3822. Unlike Arct2175FF, imidacloprid-selected Im1500FF showed 5 significantly up-regulated and zero significantly down-regulated P450s. The putative enzyme EC:1.14.14.1 (P450), coded by LL_3822, participates in 11 different metabolic pathways, including potential insecticide-resistance-related drug metabolism and xenobiotics metabolism pathways in Im1500FF [17], while this functionally important gene was significantly down-regulated by >2-fold in the Arct2175FF. All these phenomena indicate that P450s are not closely associated with detoxification and survival in permethrin-treated TPBs.

Esterases (EC 3.1) are a group of hydrolase enzymes capable of hydrolyzing compounds containing ester bonds, thereafter splitting esters into an acid and an alcohol in a chemical reaction with water [42]. Esterases are frequently implicated in the resistance of insects to organophosphates, carbamates, and pyrethroids through gene amplification, upregulation, coding sequence mutations, or a combination of these mechanisms [39]. Seven esterase genes were significantly up-regulated, and six esterase genes were significantly down-regulated in Arct2175FF. However, imidacloprid-selected Im1500FF showed nine significantly up-regulated and only one significantly down-regulated esterase gene. Of the nine up-regulated esterase genes, five (LL_699, LL_2508, LL_2600, LL_2639, and LL_L223) code for esterase ec:3.1.1.1, associated with drug metabolism and potentially resistance development in Im1500FF [17]. Only one ec:3.1.1.1-coding gene (LL-223) was found to be up-regulated in Arct2175FF, indicating that this TPB population relied less on esterases for detoxifying permethrin than for detoxifying imidacloprid.

Glutathione S-transferase (GST) utilizes glutathione to catalyze the conjugation of reduced (sulfur-substituted) glutathione, via a sulfhydryl group, to electrophilic centers on a wide variety of substrates [43]. The catalysis reactions transform a wide range of endogenous and xenobiotic compounds, including therapeutic drugs, products of oxidative stress, and pesticides, by neutralizing their electrophilic sites and rendering the products more water-soluble for further metabolization and excretion [44,45,46,47]. Several studies have indicated that GSTs play an important role in the acquisition of resistance to insecticides [46,47,48]. Results from this study showed that 5 GST genes were significantly up-regulated, while another 5 GST genes were significantly down-regulated in permethrin-treated TPBs. Pathway analysis revealed that the GST gene LL_2258, up-regulated >4.29-fold (the highest), participates in glutathione metabolism, drug metabolism, and metabolism of xenobiotics, indicating its versatilities in detoxification and importance for surviving permethrin treatment. In imidacloprid-treated TPBs, 4 GST genes were significantly down-regulated, and none was up-regulated, indicating the TPB relies less on GSTs for detoxifying imidacloprid [17].

Besides the four enzyme genes mentioned above, there are 15 other metabolic enzyme genes that were upregulated or downregulated by >2-fold in Arct2175FF. Seventeen polygalacturonase genes were up-regulated, while fourteen other polygalacturonase genes were down-regulated. Equal numbers of genes for cysteine protease and polymerase were up- and down-regulated. Five and three ligase genes were down- and up-regulated, respectively. Three- to five-fold more down-regulated genes than up-regulated genes were found in genes for amylase, lipase, hydrolase, kinase, peptidase, glucosidase, protease, and helicase. Eight-, 10.25-, and 12-fold more down-regulated than up-regulated genes were found in the genes for isomerase, cathepsin, and carboxypeptidase, respectively. Many of these genes, such as cathepsin, carboxypeptidase, and protease, are involved in the digestion of food nutrients. A significant increase in the numbers of these down-regulated enzyme-coding genes, including many genes for eggshell and vitellogenins, may be associated with the fitness cost of insecticide resistance development, resulting in a substantial decrease in colony vitality and reproduction [17]. Further investigations are needed to reveal any direct and/or indirect associations of some up-and down-regulated genes with metabolic detoxification and development of multiple and cross resistance in TPB.

## 5. Conclusions

Severe damage to crops, increased control cost, and environmental insecticide contamination would be the grievous consequences if insecticide resistance in one of the most damaging pests is left unchecked. In this study, novel microarray and extensive functional and pathway analyses of 6688 genes identified a unique set of up-regulated genes that participated in many metabolic processes and catalytic functions, confirming that metabolic resistance has evolved in TPBs from a cotton field. Oxidase and GST are the most potent detoxification enzymes, and over-expressions of these genes enable permethrin-treated TPBs to survive. Besides these commonly known detoxification enzymes, pathway analysis in this study revealed several oxidative phosphorylations associated with large number of oxidases and reductases, as well as some dehydrogenases, reductases, ATPases, and diphosphatases. These oxidative phosphorylations may directly or indirectly influence the chance of surviving TPBs after permethrin treatment. Based on all our microarray data from this study and previous studies, we found that TPB has evolved resistance to multiple insecticides, including organophosphates, neonicotinoids, and pyrethroids. Our findings indicated that the TPB population has developed a particular set of resistance genes for different insecticides. The findings of this current analysis, combined with previous data and our ongoing study on gene regulation in carbamate-treated TPB, will shed light on the resistance mechanisms of four commonly used insecticide classes and facilitate the future development of integrated strategies for monitoring and minimizing resistance risk.

## Figures and Tables

**Table 1:** Identification of 187 metabolic-enzyme-coding genes showing significantly up-regulated (≥2-fold) gene expressions in Arct2175FF resistant population using microarrays and analyzed with ArrayStar and Blat2go protocols (https://www.blast2go.com (accessed on 1 April 2023)).

Sequence ID	Length bp	Fold Increase	Fold *p* Value	Enzyme Up-Regulated	Similarity e Value	Sequence ID	Length bp	Fold Increase	Fold *p* Value	Enzyme Up-Regulated	Similarity e Value
LL_338	710	2.348	1.18 × 10^−6^	ATPase	1.48 × 10^−113^	LL_953	435	2.197	1.16 × 10^−5^	Oxidase	8.30 × 10^−80^
LL_71	656	2.143	4.57 × 10^−3^	Dehydrogenase	1.19 × 10^−16^	LL_954	408	2.369	9.09 × 10^−5^	Oxidase	4.67 × 10^−62^
LL_6549	355	4.895	2.35 × 10^−7^	Dehydrogenase	1.46 × 10^−29^	LL_963	405	5.588	2.37 × 10^−6^	Oxidase	2.95 × 10^−11^
LL_5631	461	5.12	2.47 × 10^−7^	Dehydrogenase	9.61 × 10^−28^	LL_987	702	2.25	1.45 × 10^−6^	Oxidase	4.50 × 10^−117^
LL_2370	488	8.185	2.02 × 10^−7^	Dehydrogenase	1.34 × 10^−20^	LL_1021	280	2.285	3.95 × 10^−5^	Oxidase	2.82 × 10^−35^
LL_1755	481	8.182	1.85 × 10^−7^	Dehydrogenase	1.75 × 10^−30^	LL_1022	409	5.418	4.87 × 10^−7^	Oxidase	4.72 × 10^−19^
LL_4670	373	7.505	1.94 × 10^−7^	Dehydrogenase	9.76 × 10^−30^	LL_1598	497	2.156	1.91 × 10^−6^	Oxidase	1.52 × 10^−100^
LL_187	519	5.799	4.71 × 10^−7^	Dehydrogenase	1.71 × 10^−57^	LL_1617	467	2.211	3.13 × 10^−5^	Oxidase	8.67 × 10^−89^
LL_719	500	5.756	1.93 × 10^−7^	Dehydrogenase	1.38 × 10^−57^	LL_1693	496	2.342	1.54 × 10^−5^	Oxidase	6.26 × 10^−13^
LL_4898	359	5.514	3.75 × 10^−7^	Dehydrogenase	7.74 × 10^−36^	LL_1735	490	2.974	1.07 × 10^−6^	Oxidase	2.09 × 10^−20^
LL_2943	428	3.83	1.16 × 10^−6^	Dehydrogenase	4.45 × 10^−40^	LL_1771	245	3.223	9.50 × 10^−7^	Oxidase	2.54 × 10^−11^
LL_4042	578	3.576	4.76 × 10^−7^	Dehydrogenase	3.96 × 10^−43^	LL_1819	295	2.7	2.68 × 10^−6^	Oxidase	1.13 × 10^−27^
LL_335	575	3.082	4.74 × 10^−7^	Dehydrogenase	2.85 × 10^−21^	LL_1890	343	2.68	7.02 × 10^−6^	Oxidase	2.14 × 10^−14^
LL_2478	492	2.979	6.94 × 10^−7^	Dehydrogenase	1.19 × 10^−23^	LL_1898	261	2.066	1.92 × 10^−6^	Oxidase	8.39 × 10^−25^
LL_2389	538	2.958	1.59 × 10^−6^	Dehydrogenase	2.95 × 10^−30^	LL_1918	86	4.04	7.38 × 10^−7^	Oxidase	2.00 × 10^−6^
LL_2503	402	2.422	1.02 × 10^−6^	Dehydrogenase	2.00 × 10^−16^	LL_1929	292	2.095	8.86 × 10^−6^	Oxidase	5.33 × 10^−9^
LL_50	399	2.345	1.95 × 10^−5^	Dehydrogenase	2.92 × 10^−24^	LL_2058	262	2.665	1.17 × 10^−6^	Oxidase	3.50 × 10^−8^
LL_1881	525	2.148	4.29 × 10^−5^	Dehydrogenase	1.86 × 10^−16^	LL_2203	407	7.4	3.36 × 10^−7^	Oxidase	4.99 × 10^−19^
LL_3678	375	2.124	2.36 × 10^−5^	Dehydrogenase	3.60 × 10^−20^	LL_2313	393	9.752	2.11 × 10^−7^	Oxidase	1.08 × 10^−13^
LL_3676	349	3.926	1.20 × 10^−6^	Dehydrogenase	9.11 × 10^−5^	LL_2363	409	2.602	2.94 × 10^−6^	Oxidase	8.88 × 10^−11^
LL_890	249	7.289	2.36 × 10^−5^	Dehydrogenase	6.32 × 10^−12^	LL_3734	105	4.804	1.74 × 10^−6^	Oxidase	1.30 × 10^−2^
LL_2417	414	5.058	2.58 × 10^−7^	Dehydrogenase	3.62 × 10^−11^	LL_4448	554	2.952	4.06 × 10^−6^	Oxidase	1.51 × 10^−33^
LL_258	850	3.635	4.24 × 10^−5^	Dehydrogenase	1.31 × 10^−35^	LL_4501	513	2.487	4.79 × 10^−6^	Oxidase	1.58 × 10^−10^
LL_2307	449	2.869	1.75 × 10^−6^	Dehydrogenase	4.63 × 10^−11^	LL_4995	398	2.743	1.29 × 10^−6^	Oxidase	3.25 × 10^−29^
LL_260	607	2.181	2.27 × 10^−6^	Dehydrogenase	9.19 × 10^−21^	LL_5018	565	2.43	1.46 × 10^−5^	Oxidase	5.18 × 10^−103^
LL_2319	539	2.122	3.50 × 10^−6^	Dehydrogenase	9.17 × 10^−22^	LL_5439	253	3.097	3.68 × 10^−6^	Oxidase	1.00 × 10^−6^
LL_1645	480	7.034	1.81 × 10^−7^	Dehydrogenase	4.69 × 10^−19^	LL_5548	492	4.289	4.86 × 10^−7^	Oxidase	3.00 × 10^−20^
LL_1676	308	6.319	9.62 × 10^−7^	Dehydrogenase	6.00 × 10^−18^	LL_5710	374	2.993	3.52 × 10^−6^	Oxidase	6.58 × 10^−14^
LL_36	372	6.051	2.63 × 10^−7^	Dehydrogenase	1.68 × 10^−19^	LL_6054	422	2.215	2.33 × 10^−6^	Oxidase	5.45 × 10^−85^
LL_727	344	5.971	2.34 × 10^−7^	Dehydrogenase	1.47 × 10^−15^	LL_6122	533	4.115	3.18 × 10^−7^	Oxidase	4.26 × 10^−20^
LL_1154	302	5.38	2.63 × 10^−7^	Dehydrogenase	2.05 × 10^−18^	LL_6157	457	2.397	3.52 × 10^−6^	Oxidase	3.41 × 10^−13^
LL_1608	316	7.107	8.33 × 10^−6^	Dehydrogenase	7.11 × 10^−14^	LL_6188	421	2.266	3.79 × 10^−6^	Oxidase	5.96 × 10^−33^
LL_3008	435	7.721	1.88 × 10^−7^	Dehydrogenase	1.26 × 10^−33^	LL_3359	640	7.711	2.58 × 10^−7^	P450	6.38 × 10^−45^
LL_2381	423	7.711	3.07 × 10^−7^	Dehydrogenase	5.22 × 10^−44^	LL_6649	815	2.558	1.75 × 10^−6^	Phosphatase	2.65 × 10^−10^
LL_967	621	7.652	2.18 × 10^−7^	Dehydrogenase	2.18 × 10^−42^	LL_3027	550	2.221	3.14 × 10^−6^	Phosphatase	2.15 × 10^−31^
LL_1097	254	7.562	2.71 × 10^−7^	Dehydrogenase	1.74 × 10^−11^	LL_2763	527	2.267	1.39 × 10^−5^	Phosphatase	1.44 × 10^−19^
LL_61	935	9.239	6.79 × 10^−7^	Dehydrogenase	5.15 × 10^−59^	LL_4488	495	2.121	4.24 × 10^−5^	phosphodi-Est	2.25 × 10^−83^
LL_981	525	2.182	3.05 × 10^−5^	Dehydrogenase	3.82 × 10^−26^	LL_427	465	2.061	3.38 × 10^−4^	Reductase	1.18 × 10^−15^
LL_5446	496	6.814	1.85 × 10^−7^	Dehydrogenase	2.47 × 10^−16^	LL_304	581	2.061	1.13 × 10^−4^	Reductase	4.82 × 10^−25^
LL_4956	481	6.789	3.11 × 10^−7^	Dehydrogenase	3.44 × 10^−16^	LL_5711	435	2.634	2.36 × 10^−6^	Reductase	3.16 × 10^−21^
LL_306	562	2.742	4.62 × 10^−5^	Dehydrogenase	3.11 × 10^−33^	LL_5658	520	3.26	2.94 × 10^−6^	Reductase	2.62 × 10^−19^
LL_4511	441	2.587	1.65 × 10^−6^	Dehydrogenase	6.26 × 10^−32^	LL_5691	404	11.487	1.97 × 10^−7^	Reductase	3.76 × 10^−22^
LL_506	446	2.201	1.60 × 10^−6^	Dehydrogenase	4.44 × 10^−32^	LL_431	827	5.181	5.38 × 10^−7^	Reductase	8.17 × 10^−21^
LL_2773	499	2.046	8.38 × 10^−6^	Dehydrogenase	1.51 × 10^−33^	LL_1518	286	3.235	1.92 × 10^−6^	Reductase	5.50 × 10^−6^
LL_4690	377	7.151	2.79 × 10^−7^	Dehydrogenase	3.48 × 10^−23^	LL_4317	276	3.577	1.33 × 10^−6^	Reductase	5.70 × 10^−23^
LL_1680	218	6.574	1.75 × 10^−7^	Dehydrogenase	2.87 × 10^−7^	LL_1714	371	2.219	1.80 × 10^−6^	Reductase	6.14 × 10^−4^
LL_1921	325	6.953	3.06 × 10^−7^	Dehydrogenase	2.06 × 10^−33^	LL_1719	576	5.251	9.41 × 10^−7^	Reductase	1.26 × 10^−22^
LL_690	623	3.511	4.40 × 10^−7^	Dehydrogenase	4.43 × 10^−95^	LL_2854	584	5.228	1.18 × 10^−6^	Reductase	3.79 × 10^−22^
LL_659	327	2.025	2.51 × 10^−6^	Dehydrogenase	4.66 × 10^−40^	LL_226	270	4.366	1.05 × 10^−6^	Synthase	8.24 × 10^−17^
LL_3635	716	2.341	7.77 × 10^−7^	Dehydrogenase	5.90 × 10^−43^	LL_1403	308	3.733	4.11 × 10^−7^	Synthase	1.89 × 10^−14^
LL_2244	471	6.448	4.15 × 10^−7^	Esterase	3.05 × 10^−14^	LL_1213	240	3.119	4.80 × 10^−6^	Synthase	2.52 × 10^−19^
LL_2520	557	6.444	1.99 × 10^−7^	Esterase	7.53 × 10^−31^	LL_5810	661	3.05	5.47 × 10^−7^	Synthase	7.02 × 10^−80^
LL_1233	317	4.669	1.73 × 10^−6^	Esterase	1.52 × 10^−51^	LL_407	836	2.994	1.46 × 10^−6^	Synthase	1.49 × 10^−80^
LL_2770	368	4.628	2.68 × 10^−7^	Esterase	5.28 × 10^−72^	LL_1620	677	2.713	1.35 × 10^−6^	Synthase	3.32 × 10^−76^
LL_223	668	4.468	4.99 × 10^−7^	Esterase	7.12 × 10^−45^	LL_2033	350	2.677	2.95 × 10^−6^	Synthase	8.22 × 10^−26^
LL_3979	612	3.711	6.19 × 10^−7^	Esterase	2.48 × 10^−52^	LL_6173	786	2.484	8.39 × 10^−7^	Synthase	2.66 × 10^−79^
LL_5104	610	2.02	7.61 × 10^−6^	Esterase	1.06 × 10^−139^	LL_206	648	2.448	3.48 × 10^−6^	Synthase	6.41 × 10^−59^
LL_4669	549	2.29	1.73 × 10^−5^	GST	1.60 × 10^−30^	LL_5496	234	2.384	3.67 × 10^−6^	Synthase	3.11 × 10^−24^
LL_4434	455	2.074	2.00 × 10^−3^	GST	2.75 × 10^−24^	LL_1635	574	2.351	2.10 × 10^−6^	Synthase	1.32 × 10^−60^
LL_892	412	4.92	2.25 × 10^−7^	GST	1.06 × 10^−13^	LL_1638	610	2.346	2.63 × 10^−6^	Synthase	8.66 × 10^−68^
LL_294	442	2.36	2.75 × 10^−6^	GST	1.14 × 10^−23^	LL_1655	416	2.274	9.01 × 10^−7^	Synthase	2.37 × 10^−50^
LL_2258	369	4.429	7.33 × 10^−7^	GST	2.49 × 10^−37^	LL_227	274	2.266	1.45 × 10^−6^	Synthase	2.51 × 10^−29^
LL_61	729	2.224	5.51 × 10^−6^	Oxidase	3.14 × 10^−124^	LL_225	367	2.265	1.40 × 10^−6^	Synthase	1.94 × 10^−46^
LL_95	244	2.004	1.01 × 10^−5^	Oxidase	1.98 × 10^−32^	LL_971	326	2.225	3.03 × 10^−6^	Synthase	3.48 × 10^−40^
LL_96	813	2.155	1.09 × 10^−5^	Oxidase	5.78 × 10^−130^	LL_1639	527	2.209	4.76 × 10^−6^	Synthase	2.20 × 10^−51^
LL_98	546	2.298	4.25 × 10^−6^	Oxidase	8.49 × 10^−91^	LL_2349	424	6.753	2.12 × 10^−7^	Synthase	2.37 × 10^−21^
LL_99	350	2.146	1.20 × 10^−5^	Oxidase	2.53 × 10^−55^	LL_4989	415	6.55	1.96 × 10^−7^	Synthase	1.87 × 10^−21^
LL_100	213	2.628	8.97 × 10^−6^	Oxidase	1.02 × 10^−14^	LL_6589	432	6.483	1.90 × 10^−7^	Synthase	2.52 × 10^−21^
LL_101	322	2.049	1.72 × 10^−5^	Oxidase	4.20 × 10^−34^	LL_2455	300	2.108	2.71 × 10^−6^	Synthase	4.31 × 10^−36^
LL_102	407	2.486	9.91 × 10^−6^	Oxidase	5.24 × 10^−73^	LL_6028	442	2.094	1.91 × 10^−6^	Synthase	2.11 × 10^−40^
LL_103	370	2.469	8.70 × 10^−7^	Oxidase	2.11 × 10^−64^	LL_4552	578	2.122	3.22 × 10^−6^	Synthase	1.41 × 10^−63^
LL_103	649	2.313	4.32 × 10^−5^	Oxidase	8.99 × 10^−107^	LL_3171	478	2.801	4.90 × 10^−5^	Synthase	2.13 × 10^−5^
LL_104	218	2.634	2.34 × 10^−6^	Oxidase	1.54 × 10^−28^	LL_2291	532	2.127	9.48 × 10^−6^	Synthase	2.30 × 10^−33^
LL_105	328	2.478	1.78 × 10^−6^	Oxidase	1.92 × 10^−55^	LL_3928	498	2.075	3.37 × 10^−6^	Synthase	2.55 × 10^−30^
LL_105	528	2.41	1.23 × 10^−5^	Oxidase	6.10 × 10^−91^	LL_23	497	2.073	4.67 × 10^−6^	Synthase	1.28 × 10^−36^
LL_106	668	2.459	1.08 × 10^−4^	Oxidase	3.15 × 10^−98^	LL_3406	306	2.068	7.08 × 10^−6^	Synthase	1.27 × 10^−17^
LL_107	248	2.858	1.16 × 10^−5^	Oxidase	1.55 × 10^−14^	LL_1997	403	3.3	1.23 × 10^−6^	ATPsynthase	8.75 × 10^−14^
LL_113	463	2.135	2.42 × 10^−5^	Oxidase	2.07 × 10^−86^	LL_2347	530	3.18	9.49 × 10^−7^	Synthetase	4.96 × 10^−14^
LL_117	192	2.24	6.59 × 10^−6^	Oxidase	6.00 × 10^−8^	LL_765	325	2.615	8.66 × 10^−6^	Synthetase	2.45 × 10^−32^
LL_218	697	2.256	3.80 × 10^−6^	Oxidase	3.38 × 10^−118^	LL_433	292	2.505	1.11 × 10^−5^	thio-Est	1.25 × 10^−9^
LL_219	576	2.461	1.60 × 10^−5^	Oxidase	6.78 × 10^−92^	LL_2104	677	7.393	4.80 × 10^−7^	thio-Est	7.36 × 10^−31^
LL_220	557	2.438	1.54 × 10^−5^	Oxidase	8.13 × 10^−115^	LL_5732	421	12.36	3.07 × 10^−7^	transcriptase	3.17 × 10^−32^
LL_250	275	2.096	9.44 × 10^−6^	Oxidase	5.51 × 10^−37^	LL_1753	582	4.256	4.15 × 10^−6^	Transferase	1.04 × 10^−41^
LL_376	328	2.511	2.48 × 10^−5^	Oxidase	1.90 × 10^−10^	LL_471	558	4.818	4.37 × 10^−7^	Transferase	1.17 × 10^−20^
LL_377	486	2.551	2.97 × 10^−5^	Oxidase	5.88 × 10^−13^	LL_6479	387	2.469	7.50 × 10^−6^	Transferase	5.14 × 10^−20^
LL_435	562	2.904	3.95 × 10^−6^	Oxidase	1.63 × 10^−33^	LL_3545	364	5.585	2.56 × 10^−7^	Transferase	7.38 × 10^−9^
LL_654	641	2.711	3.35 × 10^−5^	Oxidase	1.60 × 10^−112^	LL_6090	393	5.377	2.64 × 10^−7^	Transferase	8.82 × 10^−9^
LL_657	431	5.241	3.69 × 10^−7^	Oxidase	5.75 × 10^−19^	LL_394	332	2.803	7.51 × 10^−7^	Transferase	5.19 × 10^−19^
LL_700	383	6.322	1.84 × 10^−7^	Oxidase	1.69 × 10^−11^	LL_5988	638	7.09	7.10 × 10^−7^	Transferase	1.77 × 10^−13^
LL_946	311	2.248	3.56 × 10^−6^	Oxidase	1.98 × 10^−46^	LL_5562	391	2.441	9.49 × 10^−7^	Transferase	5.31 × 10^−12^
LL_947	699	2.31	4.61 × 10^−5^	Oxidase	5.95 × 10^−114^	LL_579	774	2.415	1.14 × 10^−5^	Transferase	3.81 × 10^−36^
LL_949	685	2.4	3.75 × 10^−5^	Oxidase	1.90 × 10^−113^	LL_318	580	2.042	3.11 × 10^−6^	Transferase	1.83 × 10^−34^
LL_950	597	2.268	1.17 × 10^−6^	Oxidase	8.80 × 10^−109^	LL_3864	287	2.333	1.01 × 10^−6^	Translocase	2.40 × 10^−38^
LL_951	520	2.451	4.96 × 10^−6^	Oxidase	5.72 × 10^−91^						

**Table 2:** Identification of 114 metabolic-enzyme-coding genes showing significantly down-regulated (≥2-fold) gene expressions in Arct2175FF resistant population using microarrays and analyzed with ArrayStar and Blat2go protocol (https://www.blast2go.com (accessed on 1 April 2023)).

Seq ID	Length bp	Fold Decrease	Fold *p* Value	Enzyme Down-Regulated	Similarity e Value	Seq ID	Length bp	Fold Decrease	Fold *p* Value	Enzyme Down-Regulated	Similarity e Value
LL_820	630	2.261	1.16 × 10^−5^	ATPsynthase	5.8232 × 10^−36^	LL_5255	426	6.992	1.83 × 10^−7^	Hydrolase	1.13622 × 10^−36^
LL_131	758	2.213	2.32 × 10^−5^	ATPsynthase	1.427 × 10^−34^	LL_3962	763	6.737	4.45 × 10^−7^	Hydrolase	3.50134 × 10^−73^
LL_362	634	2.209	2.30 × 10^−5^	ATPsynthase	5.1568 × 10^−36^	LL_3085	631	3.074	2.82 × 10^−4^	Hydrolase	1.07472 × 10^−63^
LL_5308	838	16.189	1.15 × 10^−4^	ATPsynthase	0	LL_4801	662	2.675	7.52 × 10^−5^	Hydrolase	1.09695 × 10^−81^
LL_1657	599	2.4	1.31 × 10^−5^	ATPsynthase	2.0737 × 10^−35^	LL_81	655	2.189	8.69 × 10^−5^	Hydrolase	1.20116 × 10^−80^
LL_5109	413	2.403	7.15 × 10^−6^	ATPsynthase	7.7674 × 10^−39^	LL_2173	584	3.073	3.68 × 10^−6^	Hydrolase	6.88239 × 10^−60^
LL_5515	433	15.49	1.87 × 10^−6^	ATPsynthase	7.9528 × 10^−52^	LL_638	851	15.847	5.86 × 10^−4^	Hydrolase	4.00172 × 10^−45^
LL_2125	645	16.045	1.50 × 10^−4^	ATPsynthase	1.037 × 10^−149^	LL_5224	359	9.698	4.73 × 10^−6^	Oxidase	4.18565 × 10^−50^
LL_2671	651	4.231	1.73 × 10^−5^	ATPsynthase	8.547 × 10^−62^	LL_759	448	2.804	1.72 × 10^−6^	Oxidase	1.89888 × 10^−52^
LL_665	530	3.644	3.39 × 10^−6^	ATPsynthase	4.9619 × 10^−47^	LL_791	562	2.931	8.69 × 10^−7^	Oxidase	2.55893 × 10^−55^
LL_3315	553	3.432	8.29 × 10^−7^	ATPsynthase	1.7271 × 10^−36^	LL_3605	368	4.275	3.64 × 10^−6^	Oxidase	4.26515 × 10^−50^
LL_1710	446	24.732	5.39 × 10^−7^	ATPsynthase	8.6728 × 10^−82^	LL_547	684	2.091	6.76 × 10^−5^	Oxidase	1.95781 × 10^−33^
LL−720	699	4.47	5.35 × 10^−7^	ATPase	1.7367 × 10^−62^	LL_567	549	3.917	5.84 × 10^−4^	Oxidase	2.22528 × 10^−19^
LL_2554	545	14.586	4.02 × 10^−7^	ATPase	5.3234 × 10^−20^	LL_5776	615	5.338	1.17 × 10^−6^	Oxidase	3.81699 × 10^−34^
LL_2418	541	2.438	3.03 × 10^−6^	ATPase	4.487 × 10^−101^	LL_746	445	17.819	1.87 × 10^−7^	P450	5.43569 × 10^−29^
LL_2337	711	2.033	7.42 × 10^−5^	ATPase	3.203 × 10^−106^	LL_4711	289	13.2	2.24 × 10^−7^	P450	5.23118 × 10^−15^
LL_3896	741	4.873	3.29 × 10^−5^	ATPase	2.145 × 10^−132^	LL_3024	834	12.933	1.42 × 10^−6^	P450	1.55029 × 10^−65^
LL_5207	595	2.259	6.53 × 10^−6^	ATPase	6.9486 × 10^−15^	LL_5133	848	6.925	1.83 × 10^−7^	P450	3.53153 × 10^−68^
LL_4647	580	13.51	2.29 × 10^−6^	ATPase	6.6458 × 10^−23^	LL_5526	242	6.463	1.96 × 10^−7^	P450	3.28538 × 10^−5^
LL_5665	608	16.92	3.05 × 10^−6^	Dehydrogenase	3.6376 × 10^−79^	LL_4607	561	2.271	1.06 × 10^−5^	P450	1.24796 × 10^−42^
LL−756	489	15.637	1.14 × 10^−4^	Dehydrogenase	5.933 × 10^−99^	LL_3822	852	2.092	5.86 × 10^−5^	P450	7.78634 × 10^−54^
LL_4889	414	3.67	7.38 × 10^−7^	Dehydrogenase	9.9234 × 10^−30^	LL_602	750	6.51	1.99 × 10^−7^	P450	1.92189 × 10^−86^
LL_3378	400	19.903	7.01 × 10^−6^	Dehydrogenase	3.7909 × 10^−41^	LL_4652	594	8.362	2.18 × 10^−7^	P450	1.13525 × 10^−46^
LL_3847	432	16.837	1.67 × 10^−6^	Dehydrogenase	4.9207 × 10^−59^	LL_4510	676	4.259	4.38 × 10^−6^	P450	1.76105 × 10^−43^
LL_6610	494	6.813	1.11 × 10^−6^	Dehydrogenase	4.0672 × 10^−61^	LL_1869	895	11.018	1.59 × 10^−5^	P450	6.25503 × 10^−97^
LL_577	484	10.593	4.94 × 10^−7^	Dehydrogenase	2.3462 × 10^−97^	LL_4067	527	3.524	1.07 × 10^−6^	Peroxidase	2.14959 × 10^−68^
LL_5205	489	35.097	7.93 × 10^−7^	Dehydrogenase	9.053 × 10^−45^	LL_2533	678	5.796	2.33 × 10^−6^	Phosphate synthase	7.8801 × 10^−81^
LL_6051	522	24.257	1.17 × 10^−6^	Dehydrogenase	1.1249 × 10^−44^	LL_4724	569	3.001	1.30 × 10^−6^	Reductase/Peptidase	2.53791 × 10^−66^
LL_2340	753	12.215	1.62 × 10^−5^	Dehydrogenase	8.624 × 10^−148^	LL_268	440	2.082	7.69 × 10^−6^	Reductase/Peptidase	2.5354 × 10^−42^
LL_4467	758	2.004	3.99 × 10^−6^	Dehydrogenase	4.0349 × 10^−51^	LL_5613	882	10.058	2.94 × 10^−6^	Reductase/Peptidase	3.3655 × 10^−134^
LL_3786	724	13.953	2.99 × 10^−4^	Dehydrogenase	1.718 × 10^−82^	LL_379	542	14.788	2.01 × 10^−4^	Reductase	4.80605 × 10^−35^
LL_266	577	2.258	3.83 × 10^−6^	Dehydrogenase	5.5356 × 10^−42^	LL_3192	636	32.861	1.02 × 10^−4^	Reductase	1.13827 × 10^−84^
LL_3436	670	10.494	4.49 × 10^−7^	Dehydrogenase	1.3348 × 10^−56^	LL_5391	501	22.722	8.77 × 10^−7^	Reductase	5.91618 × 10^−36^
LL−666	466	36.377	3.27 × 10^−5^	Dehydrogenase	5.0621 × 10^−45^	LL_5648	333	2.429	1.73 × 10^−6^	Reductase	1.39958 × 10^−37^
LL_2979	655	2.278	2.60 × 10^−6^	Dehydrogenase	1.4154 × 10^−38^	LL_4420	467	2.409	4.83 × 10^−4^	Reductase	4.50782 × 10^−23^
LL−566	715	18.171	3.05 × 10^−6^	Dehydrogenase	8.2028 × 10^−79^	LL_2358	722	5.533	9.06 × 10^−7^	Reductase	6.72123 × 10^−60^
LL−343	667	11.421	3.59 × 10^−4^	Dehydrogenase	2.51 × 10^−128^	LL_202	989	3.371	4.49 × 10^−7^	Reductase	2.685 × 10^−117^
LL_3297	846	4.133	6.27 × 10^−7^	Dehydrogenase	1.668 × 10^−149^	LL_3600	591	2.256	2.75 × 10^−5^	Reductase	6.34142 × 10^−81^
LL_3901	397	22.258	1.41 × 10^−6^	Dehydrogenase	3.1323 × 10^−34^	LL_5172	608	12.781	1.48 × 10^−5^	Reductase	2.30481 × 10^−8^
LL_3798	502	2.541	1.68 × 10^−6^	Dehydrogenase	5.2771 × 10^−98^	LL_3614	583	9.678	5.35 × 10^−7^	Transferase	2.08268 × 10^−51^
LL_3511	451	3.334	2.06 × 10^−6^	Dehydrogenase	8.5979 × 10^−12^	LL_3257	560	10.475	9.42 × 10^−6^	Transferase	7.80857 × 10^−66^
LL_1707	490	7.033	3.80 × 10^−7^	Dehydrogenase	8.0481 × 10^−28^	LL_2855	691	6.424	2.71 × 10^−6^	Transferase	4.25986 × 10^−36^
LL_5362	778	3.071	5.35 × 10^−6^	Dehydrogenase	5.9194 × 10^−81^	LL_3577	658	6.581	2.99 × 10^−7^	Transferase	1.00583 × 10^−60^
LL_5724	544	2.054	5.44 × 10^−6^	Dehydrogenase	2.8814 × 10^−37^	LL_6052	669	5.969	3.22 × 10^−5^	Transferase	2.22505 × 10^−67^
LL_713	739	4.58	6.67 × 10^−5^	Dehydrogenase	1.094 × 10^−112^	LL_5629	417	17.012	3.67 × 10^−7^	Transferase	4.18095 × 10^−18^
LL_699	788	5.995	2.68 × 10^−3^	Esterase	0	LL_612	646	12.492	3.95 × 10^−7^	Transferase	2.42378 × 10^−32^
LL_2508	864	4.172	1.03 × 10^−6^	Esterase	9.7275 × 10^−60^	LL_3986	639	2.74	1.58 × 10^−5^	Transferase	4.9962 × 10^−26^
LL_4010	442	2.276	7.49 × 10^−7^	Esterase	7.7999 × 10^−15^	LL_6404	503	2.385	2.71 × 10^−6^	Transferase	2.58015 × 10^−9^
LL_6522	772	4.711	2.07 × 10^−5^	Esterase	5.4832 × 10^−60^	LL_2846	444	23.112	7.11 × 10^−7^	Transferase	8.4084 × 10^−64^
LL_2193	811	3.424	3.58 × 10^−4^	Esterase	2.19 × 10^−60^	LL_2350	626	3.107	2.23 × 10^−6^	Transferase	1.41107 × 10^−22^
LL_227	422	2.86	1.95 × 10^−6^	Esterase	2.9057 × 10^−8^	LL_5050	537	2.661	1.20 × 10^−6^	Transferase	7.62917 × 10^−90^
LL_6284	676	5.838	9.42 × 10^−7^	GST	1.4326 × 10^−98^	LL_2224	825	5.441	2.30 × 10^−7^	Transferase	6.54044 × 10^−41^
LL_671	615	17.462	5.11 × 10^−6^	GST	9.2649 × 10^−41^	LL_6630	335	3.937	3.54 × 10^−6^	Transferase	2.36292 × 10^−21^
LL_142	422	4.171	2.82 × 10^−6^	GST	1.8419 × 10^−35^	LL_2365	515	18.72	1.71 × 10^−6^	Transferase	3.40881 × 10^−20^
LL_4847	625	2.158	6.43 × 10^−6^	GST	1.2083 × 10^−46^	LL_3388	813	15.797	4.11 × 10^−6^	Transferase	1.64364 × 10^−25^
LL_6603	532	3.931	1.84 × 10^−6^	GST	1.6255 × 10^−25^	LL_803	126	3.401	3.03 × 10^−6^	Transferase	1.11026 × 10^−10^
LL_1768	880	27.816	2.92 × 10^−6^	Hydrolase	1.2626 × 10^−80^	LL_732	401	3.84	5.48 × 10^−7^	Transferase	1.32524 × 10^−67^

**Table 3:** Up-regulation is shown for Arct2175FF and relevant genes’ major roles in metabolic pathways (KEGG analysis www.blast2go.com (https://www.blast2go.com (accessed on 1 April 2023)).

Pathway	Seqs in Pathway	Seqs of Enzyme	Sequence ID	Enzyme	Enzyme ID
Oxidative phosphorylation	68	37	LL_95, LL_96, LL_98, LL_99, LL_100, LL_101, LL_102, LL_103, LL_104, LL_105, LL_106, LL_107, LL_218, LL_219, LL_220, LL_250, LL_946, LL_947, LL_949, LL_950, LL_951, LL_953, LL_954, LL_987, LL_1021, LL_1598, LL_1617, LL_1819, LL_1898, LL_1929, LL_5018, LL_6054, LL-61, LL-103, LL-105, LL-113, LL-654	oxidase	ec:1.9.3.1
Oxidative phosphorylation	68	23	LL_36, LL_61, LL_258, LL_260, LL_727, LL_890, LL_967, LL_981, LL_1097, LL_1154, LL_1608, LL_1645, LL_1676, LL_1680, LL_1921, LL_2307, LL_2319, LL_2381, LL_2417, LL_2943, LL_3008, LL_4690, LL-690	reductase (H+-translocating)	ec:1.6.5.3
Oxidative phosphorylation	68	2	LL_50, LL_2389	dehydrogenase	ec:1.6.99.3
Oxidative phosphorylation	68	4	LL_435, LL_2313, LL_4448, LL_4995	reductase	ec:1.10.2.2
Oxidative phosphorylation	68	1	LL_1997	ATPase	ec:3.6.3.6
Oxidative phosphorylation	68	1	LL_3027	diphosphatase	ec:3.6.1.1
Glutathione metabolism	1	1	LL_2258	transferase	ec:2.5.1.18
Drug metabolism—cytochrome P450	1	1	LL_2258	transferase	ec:2.5.1.18
Metabolism of xenobiotics by cytochrome P450	1	1	LL_2258	transferase	ec:2.5.1.18

**Table 4:** Down-regulation is shown for Arct2175FF and relevant genes’ major roles in metabolic pathways (KEGG analysis www.blast2go.com (https://www.blast2go.com (accessed on 1 April 2023)).

Pathway	Seqs in Pathway	Seqs of Enzyme	Sequence ID	Enzyme	Enzyme ID
Glutathione metabolism	4	1	LL_577	dehydrogenase (NADP+)	ec:1.1.1.42
Glutathione metabolism	4	1	LL_5224	glutathione peroxidase	ec:1.11.1.12
Glutathione metabolism	4	2	LL_4067, LL_5224	peroxidase	ec:1.11.1.9
Glutathione metabolism	4	1	LL_6284	transferase	ec:2.5.1.18
Oxidative phosphorylation	24	2	LL_791, LL-759	oxidase	ec:1.9.3.1
Oxidative phosphorylation	24	2	LL_3847, LL_6610	dehydrogenase (ubiquinone)	ec:1.3.5.1
Oxidative phosphorylation	24	3	LL_268, LL_4724, LL_5613	reductase	ec:1.10.2.2
Oxidative phosphorylation	24	12	LL_131, LL_820, LL_1657, LL_1710, LL_2125, LL_2671, LL_3315, LL_5109, LL_5308, LL_5515, LL-362, LL-665	ATPase	ec:3.6.3.6
Oxidative phosphorylation	24	5	LL_3378, LL_4889, LL_5665, LL-379, LL-756	reductase (H+-translocating)	ec:1.6.5.3
Drug metabolism—other enzymes	1	1	LL_2533	phosphoribosyltransferase	ec:2.4.2.10
Drug metabolism—cytochrome P450	1	1	LL_6284	transferase	ec:2.5.1.18
Metabolism of xenobiotics by cytochrome P450	1	1	LL_6284	transferase	ec:2.5.1.18

## Data Availability

All data were provided in the publication.
